# Ventriculostomy-related intracranial hemorrhage following surgical and endovascular treatment of ruptured aneurysms

**DOI:** 10.1007/s10143-022-01777-5

**Published:** 2022-04-29

**Authors:** Moritz Lenschow, Niklas von Spreckelsen, Sergej Telentschak, Christoph Kabbasch, Roland Goldbrunner, Stefan Grau

**Affiliations:** 1 Center for Neurosurgery, University Hospital of Cologne, Kerpenerstr. 62, 50937 Cologne, Germany; 2 Department of Neuroradiology, University Hospital of Cologne, Kerpenerstr. 62, 50937, Cologne, Germany

**Keywords:** Aneurysm, Subarachnoid hemorrhage, Hydrocephalus, Complication, Intracerebral hemorrhage

## Abstract

Endovascular therapy of ruptured aneurysms is regularly accompanied by periprocedural heparinization and requires the use of periprocedural antiplatelets in more complex cases. This raises concerns regarding increased bleeding risks in the case of frequently required ventriculostomy. The aim of this study was to analyze risk factors for ventriculostomy-related intracranial hemorrhages (VS-ICH) in endovascular or surgical treatment of ruptured aneurysms with a focus on antithrombotic therapy. In this retrospective analysis, we included patients admitted to our institution over a 12-year period who had received at least one ventriculostomy due to subarachnoid hemorrhage-related hydrocephalus. Patients were dichotomized into an endovascular and surgical group and rates of VS-ICH were compared. Risk factors for VS-ICH were assessed in uni- and multivariate analyses. A total of 606 ventriculostomies were performed in 328 patients. Within the endovascular group, antiplatelet therapy was used in 44.8% of cases. The overall rate of ventriculostomy-related intracranial hemorrhage was 13.1%. Endovascular treatment was associated with a higher rate of VS-ICH compared to surgical treatment (*p* = 0.011), but not in cases without antiplatelet therapy (*p* = 0.166). Application of any antiplatelet therapy (odds ratio, 2.647 [95% confidence interval, 1.141–6.143]) and number of ventriculostomies (odds ratio, 2.513 [95% confidence interval, 1.859–3.395]) were independent predictors of ventriculostomy-related hemorrhages. Our findings indicate an increased risk of VS-ICH in the endovascular group if administration of antiplatelets was required. While this aspect has to be included into treatment decision-making, it must be weighed against the benefits of endovascular techniques.

## Introduction

Treatment of ruptured aneurysms is generally based on an interdisciplinary consensus. While the feasibility of surgery depends on the clinical status and aneurysm location, recent improvements in endovascular devices have widened the spectrum of endovascular treatment [4]. However, endovascular aneurysm treatment is often accompanied by intraprocedural heparinization, and intravascular devices such as microstents or flow-diverters also require peri- and post-procedural administration of single or dual antiplatelets [17, 21].

**Fig. 1:**
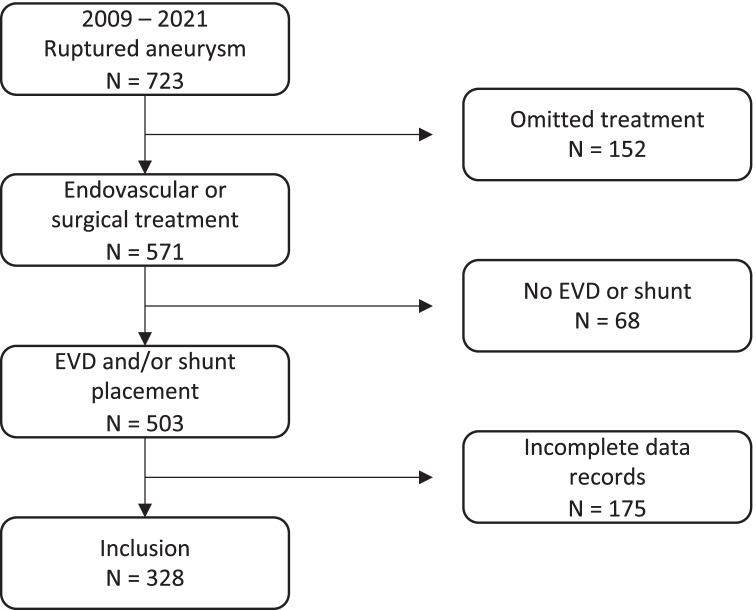
Ventriculostomy prior to endovascular aneurysm treatment necessitating permanent antiplatelet therapy (left) and VS-ICH in the same patient following a subsequent catheter exchange (right)

In contrast to elective aneurysm treatment, subarachnoid hemorrhage often requires emergency ventriculostomy as well as consecutive ventriculostomies in the case of dysfunction or due to chronic hydrocephalus [22]. While the relevance of antithrombotic therapy for ventriculostomy-related intracranial hemorrhage (VS-ICH) in the setting of acutely ruptured aneurysm has been previously reported, data comparing the risk of VS-ICH between endovascular and surgical treatment are sparse, and most studies have not considered patients with multiple ventriculostomies (Fig. 1) [2, 3].

The aim of this study was to identify risk factors for VS-ICH in a large cohort comprising endovascular and surgical aneurysm treatment.

## Materials and methods

### Data collection

**Fig. 2:**
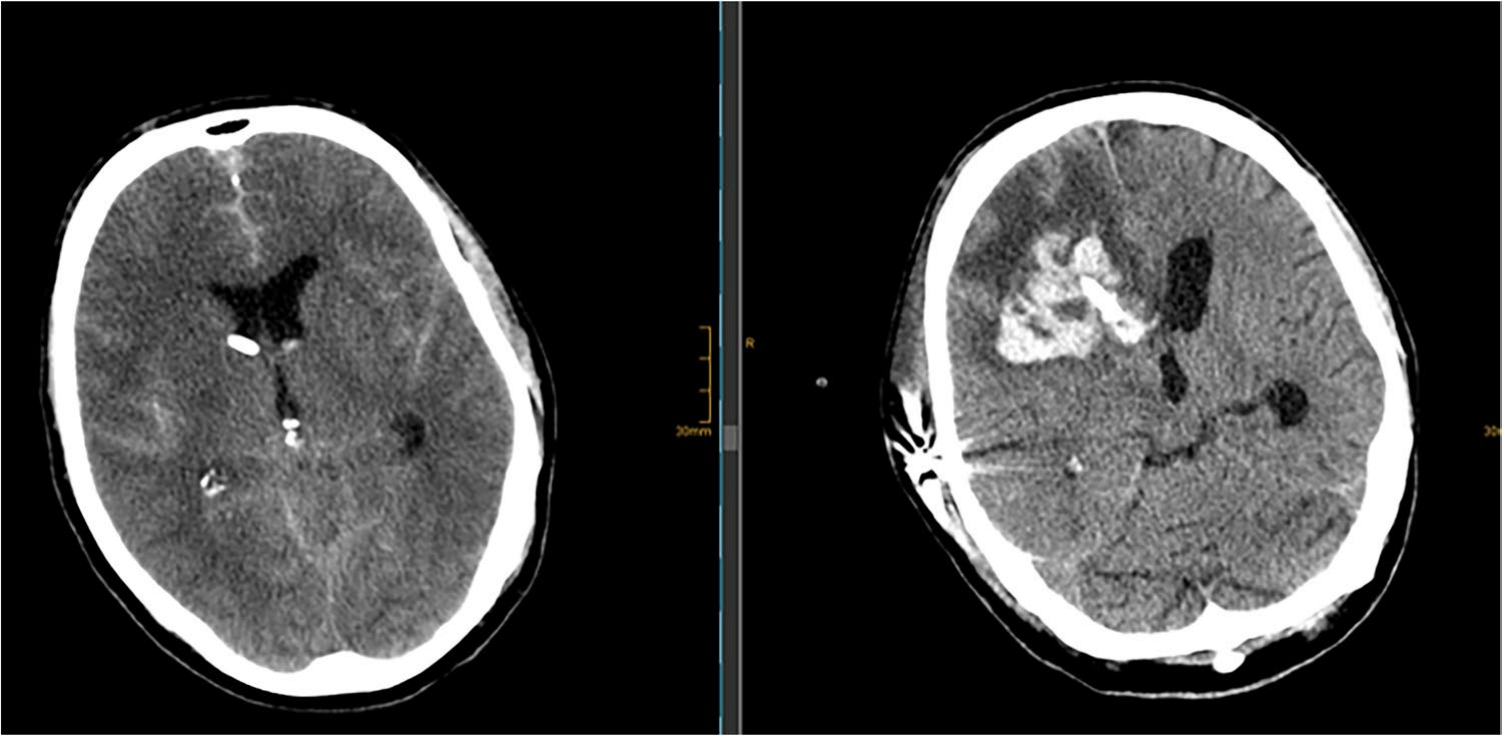
Flow chart of the study population

In this retrospective analysis, we included all consecutive patients admitted to our institution between March 2009 and March 2021 who received at least one ventriculostomy due to subarachnoid hemorrhage-related hydrocephalus. Patients were excluded in the case of missing data and in cases when aneurysm treatment was omitted (e.g., primary palliative cases; see Fig. 2).

The following parameters were recorded: treatment modality, age, sex, aneurysm location, number and type of ventriculostomies (external ventricular drain or shunt), endovascular technique, type, and timing of antiplatelets used as well as heparin administration [7, 12]. Hunt and Hess grade was determined based on the initial examination at the time of admission to the emergency department, either at our center or at the primary treating hospital if patients were transferred. Accordingly, the modified Fisher score was assessed based on the initial imaging at our center or the transferring hospital.

Individual treatment strategies were determined in an interdisciplinary case review by a senior neuro-radiologist and a senior vascular neurosurgeon. Accordingly, patients were categorized into a surgical and an endovascular group.

Study approval was obtained by the local ethics committee (reference number: 21–1262). Written patient consent was not required.

### Antithrombotic drugs

Intra- and post-procedural antiplatelet drugs as well as intra -procedural systemic heparinization were administered based on a case-by-case decision at the discretion of the treating neuro-radiologist.

Prophylactic anticoagulation (aPTT < 35) using intravenous unfractionated heparin was administered in all cases during the course of the ICU stay. Preexisting oral anticoagulants were discontinued following the diagnosis of subarachnoid hemorrhage.

### Ventriculostomy

Ventriculostomies included placement of external ventricular drains and ventricular catheters for ventriculoperitoneal shunting. An external ventricular drainage was placed when increased intracranial pressure was clinically suspected (e.g., Hunt and Hess grade ≥ 3) and/or when there was radiologic evidence of hydrocephalus (ballooning of the frontal horns of the lateral ventricles and/or third ventricle or size of the temporal horns ≥ 2 mm) [17]. Ventriculo-peritoneal shunts were placed following at least one failed weaning attempt and no earlier than 21 days after aneurysm rupture. For repeat ventriculostomies, the preexisting puncture channel was used unless progressive unilateral intraventricular hemorrhagic obstruction required a contralateral approach.

All external ventricular drains were placed in a bedside procedure in the angiography suite or the intensive care unit; ventriculo-peritoneal shunts were placed in the surgical suite. Ventricular catheters were usually passed into the frontal horn of the lateral right ventricle following burr hole placement at the Kocher’s point, with procedural adjustments according to the case-specific conditions.

### Determination of ICH

Hemorrhagic complications were assessed using follow-up CT scans conducted for all patients during their clinical course. A routine imaging was obtained within 24 h following initial ventriculostomy as well as prior to drain removal or shunt placement (clamp trial). In cases of ventriculoperitoneal shunt placement, a CT scan was routinely obtained within 24 h postoperatively; after removal of the external ventricular drain, follow-up imaging was not routinely performed. In cases of external ventricular drain revision, pre- and postoperative imaging was obtained on a case-by-case basis (e.g., no routine imaging in case of uncomplicated drain replacement due to ventriculitis). Further CT scans were performed between days 5 and 7 and days 11 and 14 following aneurysm rupture as well as in case of neurological deterioration or suspected vasospasm.

In the case of a VS-ICH, hematoma volume was calculated using the volumetric tool function of the inomed Planning System (iPS—inomed Planning System; Version 6) software. Catheter volumes were subtracted.

### Statistical analysis

Baseline characteristics are displayed using descriptive statistics. Categorical variables were compared by a chi-square and a Fisher’s exact test, when appropriate. Continuous variables were tested for normal distribution using the Kolmogorov–Smirnov test. Group means from normally distributed data were compared using a two-sided unpaired Student’s *t*-test while a Mann–Whitney *U* test was used for non-normally distributed data. Factors significantly associated with ICH in the univariate analysis were included in a binary logistic stepwise regression model to identify independent risk factors for ventriculostomy-related ICH.

All calculations were performed using SPSS software (Version 27, IBM SPSS Statistics for Windows, Armonk, NY, USA). A *p*-value < 0.05 was considered statistically significant.

## Results

### Patient characteristics

We included 328 patients with aneurysmal subarachnoid hemorrhage who received ventriculostomies due to hydrocephalus between March 2009 and March 2021.

Aneurysms were treated endovascularly in 210 (64.0%) patients or by microsurgical clipping in 118 (36.0%) patients. The median patient age was 56 (range 15 to 89) years; 63.7% of all patients were female. Median Hunt and Hess grade was 3 (range 1 to 5) and median-modified Fisher score was 3 (range 1 to 4). Most aneurysms were located at the anterior communicating (36.0%) and middle cerebral artery (22.3%). The location of treated aneurysms significantly differed between the surgical and endovascular group (*p* < 0.001). The groups did not differ with respect to age (*p* = 0.263), gender (*p* = 0.964), Hunt and Hess grade (*p* = 0.073), and modified Fisher score (*p* = 0.968). A total of 606 ventriculostomies were performed (506 external ventricular drains and 100 shunting procedures). The surgical and endovascular group did not differ with regard to the total number of ventriculostomies (*p* = 0.319) or the number of ventriculostomies per patient (*p* = 0.685). Ventriculostomy prior to treatment of the ruptured aneurysm was significantly more frequent in the endovascular group than in the surgical group (79.5 vs. 62.7%; *p* = 0.001). Median platelet count in the overall cohort was 220 (range 87 to 553). A platelet count less than 100,000 at initial presentation was observed in one patient of the surgical group (0.8%) and none in the endovascular group (*p* = 0.360). Median International normalized ratio (INR) at initial presentation in the study group was 1 (range 0.9 to 3.6) and did not significantly differ between the surgical (1.0, range 0.9 to 3.6) and endovascular (1.0, range 0.9 to 3.5) group (*p* = 0.616). The number of patients with an elevated INR (INR > 1.1) at initial presentation did not differ significantly between the surgical (5.9%) and endovascular (4.8%) group (*p* = 0.616). Detailed patient characteristics are listed in Table 1.

**Table 1:** Patient characteristics (*n*, column %)

	All patients (*n* = 328)	Endovascular group (*n* = 210)	Surgery group (*n* = 118)	*P*-value
Age (median, range)	56 (15–89) years	57 (15–89) years	55 (25–86) years	0.263
Gender				0.964
Female	209 (63.7%)	134 (63.8%)	75 (63.6%)	
Male	119 (36.3%)	76 (35.2%)	43 (36.4%)	
Hunt & Hess grade				0.073
1	17 (5.2%)	8 (3.8%)	9 (7.6%)	
2	87 (26.5%)	55 (26.2%)	32 (27.1%)	
3	80 (24.4%)	46 (21.9%)	34 (28.8%)	
4	118 (36.0%)	83 (39.5%)	35 (29.7%)	
5	26 (7.9%)	18 (8.6%)	8 (6.8%)	
Modified Fisher score ^1^				0.968
1	50 (15.8%)	22 (19.6%)	28 (13.7%)	
2	28 (8.8%)	11 (9.8%)	17 (8.3%)	
3	93 (29.3%)	23 (20.5%)	70 (34.1%)	
4	146 (46.1%)	56 (50.0%)	90 (43.9%)	
Location				< 0.001
Anterior cerebral artery	14 (4.3%)	13 (6.2%)	1 (0.8%)	
Anterior com. artery	118 (36.0%)	80 (38.1%)	38 (32.2%)	
Middle cerebral artery	73 (22.3%)	19 (9.0%)	54 (45.5%)	
Internal carotid artery	31 (9.5%)	23 (11.0%)	8 (6.8%)	
Posterior com. artery	31 (9.5%)	21 (10.0%)	10 (8.5%)	
Posterior cerebral artery	8 (2.4%)	6 (2.9%)	2 (1.7%)	
Basilar/vertebral artery	53 (16.2%)	48 (22.9%)	5 (4.2%)	
International normalized ratio (median, range) at initial presentation	1 (0.9–3.6)	1 (0,9–3,5)	1.0 (0.9–3.6)	0.563
Platelet count (median, range) at initial presentation	220 (87–553)	224 (100–553)	218 (87–522)	0.165
Any antiplatelets at initial presentation	22 (6.7%)	14 (6.7%)	8 (6.8%)	0.969
Oral anticoagulation at initial presentation	19 (5.8%)	8 (6.8%)	11 (5.2%)	0.566
Timing of ventriculostomy				
Pre-treatment	241 (73.5%)	167 (79.5%)	74 (62.7%)	0.001
Post-treatment	214 (65.2%)	133 (63.3%)	81 (68.6%)	0.332
Total no. of ventriculostomies	606	395	211	0.319
No. of ventriculostomies per patient				0.685
1–2	259 (79.9%)	164 (78.1%)	95 (89,5%)	
3–4	57 (17.4%)	39 (18.6%)	18 (15.3%)	
≥ 5	12 (3.7%)	7 (3.3%)	5 (4.2%)	

^1^Not available in 11 cases

### Antiplatelet and anticoagulation therapy

No antiplatelets or perioperative heparin was administered in the surgical group. Within the endovascular group, antiplatelets and heparin were periprocedurally administered as part of the endovascular treatment in 43.3% and 53.8% of all cases, respectively. Acetylsalicylic acid was used in 42.9% of all endovascular cases with an intra-procedural loading dose in 77.8% of these cases (mean 450 ± 120.4 mg) and a post-procedural oral maintenance dose of 100 mg. Clopidogrel was used in 27.1% of all endovascular cases with an intra-procedural loading dose (mean 311.0 ± 70.3 mg) in 70.7%. The oral maintenance dose was 75 mg in 57 cases and 150 mg in one case (due to a known low responder to clopidogrel). Platelet reactivity to antiplatelet medications was not routinely measured. Tirofiban was used intra- and post-procedural in 26.7% of all endovascular cases; dosing was based on the patient’s weight. Intra-procedural anticoagulation using intravenous unfractionated heparin was administered in 53.8% of all endovascular cases; the mean dosage was 4106.8 ± 1546.4 I.U.

### Endovascular technique

Coil embolization was conducted in 68.1% and stent-assisted coiling in 21.4% of all endovascular cases. A Woven EndoBridge device or flow-diverter was used in 3.3% and 7.1% of cases, respectively. Table 2 provides an overview of the techniques and medications used in the endovascular group.

**Table 2:** Techniques and medications used in the endovascular group (*n*, column %)

	Endovascular patients (*n* = 210)
Techniques	
Coil embolization	143 (68.1%)
Stent-assisted coiling	45 (21.4%)
Woven EndoBridge device	7 (3.3%)
Flow-diverter stent	15 (7.1%)
Antiplatelet agents (intra- and/or postprocedural administration)	
Acetylsalicylic acid	90 (42.9%)
Clopidogrel	57 (27.1%)
Tirofiban	54 (25.7%)
Antiplatelet and anticoagulation agents	
Any antiplatelets ^1^	92 (43.8%)
Heparin	113 (53.8%)
Neither antiplatelets nor heparin	59 (28.1%)
Single vs. dual oral antiplatelet therapy	
Single oral antiplatelet (acetylsalicylic acid)	33 (15.7%)
Dual oral antiplatelets (acetylsalicylic acid + clopidogrel)	57 (27.1%)

^1^Either acetylsalicylic acid or clopidogrel or tirofiban

### Ventriculostomy-related ICH

VS-ICH occurred in 43 patients (13.1%); among these, 10 (23.3%) showed intraventricular hemorrhage. The mean hematoma volume was 1.4 ± 6.5 ml. VS-ICH was significantly more frequent in the endovascular group (16.7 vs 6.8%, *p* = 0.011). Mean hematoma size was significantly larger in the endovascular group (1.8 ± 7.6 vs. 0.9 ± 3.4 ml; *p* = 0.011). Occurrence of intraventricular hemorrhage did not differ between the two groups (1.7 vs. 3.8%; *p* = 0.341). None of the VS-ICH the study population was evacuated surgically. Detailed information is provided in Table 3.

**Table 3:** Hemorrhagic complications (*n*, column %)

	All patients (*n* = 328)	Endovascular group (*n* = 210)	Surgery group (*n* = 118)	*P*-value
Hemorrhagic events	43 (13.1%)	35 (16.7%)	8 (6.8%)	0.011
Hematoma size in ml (mean, standard deviation)	1.92 (± 9.92) ml	2.44 (± 11.66) ml	0.99 (± 5.56) ml	0.012
Intraventricular hemorrhage	10 (3.0%)	8 (3.8%)	2 (1.7%)	0.341

### Risk factors for ventriculostomy-related ICH

In the entire cohort, heparin administration (*p* = 0.150), single vs. double oral antiplatelets (*p* = 0.163), age (*p* = 0.389), gender (*p* = 0.496), Hunt and Hess grade (*p* = 0.229), modified Fisher score (*p* = 0.098), and timing of ventriculostomy (*p* = 0.356) did not impact the rate of VS-ICH.

In univariate analysis, the administration of any antiplatelet drug (acetylsalicylic acid, clopidogrel, and/or tirofiban, *p* = 0.002) and the number of ventriculostomies (*p* < 0.001) were significantly associated with VS-ICH, while for patients not receiving antiplatelet drugs, there was no statistical difference in the rate of VS-ICH comparing surgical and endovascular treatment (8/118, 6.8%, vs. 14/116, 12.1%; *p* = 0.166).

Mean hematoma volume was greater in patients receiving any antiplatelet drug compared to those without (3.4 ± 14.6 vs. 1.4 ± 7.3 ml; *p* = 0.001). The risk for intraventricular hemorrhage was not associated with the administration of antiplatelet drugs (5/94, 5.3%, vs. 5/234, 2.1%; *p* = 0.157). Timing of ventriculostomy in the subgroup of patients on antiplatelet drugs was not associated with an increased risk of VS-ICH (5/38, 13.2% pre-occlusion ventriculostomy, vs. 2/18, 11.1% post-occlusion ventriculostomy; *p* = 1.000). In multivariate regression analysis, the administration of any antiplatelet drug (odds ratio [OR], 2.647 [95% confidence interval [CI], 1.141 to 6.143]) and number of ventriculostomies (OR, 2.513 [95% CI, 1.859 to 3.395]) remained independent factors significantly associated with VS-ICH. Detailed information regarding risk factors for VS-ICH is displayed in Tables 4 and 5.

**Table 4:** Predictive factors for ventriculostomy-related hemorrhagic events (*n*, row %)

	Hemorrhagic event (*n* = 43)	No hemorrhagic event (*n* = 285)	*P*-value
Any oral or intravenous antiplatelets	21 (22.8%)	71 (77.2%)	0.002
No antiplatelets or anticoagulation agents	17 (9.6%)	160 (90.4%)	0.042
Heparin administration	19 (16.8%)	94 (83.2%)	0.150
Single vs. dual oral antiplatelets			0.163
Single oral antiplatelet	5 (15.2%)	28 (84.8%)	
Double oral antiplatelets	16 (28.1%)	41 (71.9%)	
Age (median, range)	57 (36–80) years	56 (15–89) years	0.389
Gender			0.496
Female	25 (12.0%)	184 (88.0%)	
Male	18 (15.1%)	101 (84.9%)	
Hunt & Hess grade			0.229
1	0 (0.0%)	17 (100%)	
2	8 (9.2%)	79 (90.8%)	
3	15 (18.8%)	65 (81.3%)	
4	17 (14.4%)	101 (85.6%)	
5	3 (11.5%)	23 (88.5%)	
Modified Fisher score			0.098
1	0 (0.0%)	50 (100%)	
2	5 (17.9%)	23 (82.1%)	
3	16 (17.2%)	77 (82.8%)	
4	22 (15.1%)	124 (84.9%)	
Timing of ventriculostomy ^1^			0.356
Pre-treatment	8 (7.0%)	106 (93.0%)	
Post-treatment	3 (3.4%)	84 (96.6%)	
Number of ventriculostomies (mean, standard deviation)	3.3 (2.4)	1.6 (0.9)	< 0.001
Number of ventriculostomies per patient			< 0.001
1–2	21 (8.1%)	238 (91.9%)	
3–4	12 (21.1%)	45 (78.9%)	
≥ 5	10 (83.3%)	2 (16.7%)	

^1^Cases with both pre- and post-treatment ventriculostomy not included (*n* = 127)

**Table 5:** Binary logistic regression model

	Odds ratio (95% confidence interval)
Any antiplatelets	2.647 (1.141–6.143)
Number of ventriculostomies	2.513 (1.859–3.395)
Endovascular treatment	2.219 (0.777–6.333)

## Discussion

Although ruptured aneurysms are increasingly amenable to modern endovascular techniques, antiplatelet and heparin administration are frequently required, thus raising concerns regarding ventriculostomy-related ICH.

In this series, VS-ICH occurred in 13.1% of all cases, which is in line with other studies reporting rates from 10 to 20% [3, 10]. While the observed predominance of ICH in the endovascular group confirms previously reported data from Scheller et al., this difference was no longer present among patients receiving no antiplatelet therapy [20]. Accordingly, the major finding of this study is that the administration of any antiplatelet therapy was the most prominent risk factor for VS-ICH.

Previous studies reporting on the impact of dual antiplatelet therapy on VS-ICH showed controversial results: while some authors reported an increased risk for ventriculostomy-related ICH after peri- and postprocedural administration of acetylsalicylic acid and clopidogrel (ranging from 7 to 63%), others could not demonstrate any correlation between dual antiplatelet therapy and VS-ICH [1, 3, 10, 11, 14], and data elucidating the impact of specific substances are sparse. Only one study compared the administration of acetylsalicylic acid and clopidogrel, defining both as a risk factor for VS-ICH [7]. While some reports identified tirofiban as a risk factor for VS-ICH [2, 18], this could not be confirmed by other studies [13, 23, 24]. Of note, the number of ventriculostomies in these studies was either low or even neglected. The heterogeneous cohort and consequently small subgroups in the present study did not allow a sound statistical evaluation of the individual antiplatelet agents. To answer this question, larger, multicenter studies are warranted.

Periprocedural heparin administration had no effect on the rate of VS-ICH, consistent with the largely homogeneous results of previous studies [2, 9, 14, 15, 22]. Conflicting results may be explained by different time intervals between ventriculostomy and heparinization [8].

In general, conflicting results with regard to antithrombotic medication may be explained by statistical limitations and differences in study design; also, dosage regimes are not standardized and rely on local standards, resulting in variable loading and maintenance dosages. Furthermore, non-responder rates of up to 40% may limit the explanatory power of these analyses [3, 4, 7].

The issue of ventriculostomy timing in relation to anticoagulation treatment has been controversially discussed in literature, in particular addressing the question of whether the initiation of antiplatelet therapy shortly after or prior to ventriculostomy is safer. Performing the ventriculostomy before starting antiplatelet therapy has been recommended based on reports of a higher incidence of ICH in case of subsequent ventriculostomy [5, 17, 19, 21]. Indeed, in this study, ventriculostomy prior to aneurysm occlusion was significantly more frequent in the endovascular group, despite comparable clinical status, which may be explained by a more deliberate indication for prophylactic external ventricular drain placement in order to avoid ventriculostomy under antiplatelet therapy. However, our data did not show an impact of ventriculostomy timing on bleeding rates, neither in the overall population nor the subgroup on antiplatelet drugs, which is in line with the results of two recent studies [16, 18].

While most studies report VS-ICH rates following a single ventriculostomy, this does not necessarily reflect the clinical reality since many patients require subsequent ventriculo-peritoneal shunting or their device exchanged due to obstruction or infection. Consequently, the number of ventriculostomies by far exceeded the number of patients in this study. Since each surgical procedure implies a separate surgical risk, the aspect of repeated surgery must not be neglected in this patient group. In this present study, the number of ventriculostomies was a significant risk factor for VS-ICH in both uni- and multivariate analysis. Considering that previous studies frequently ignore this issue, the actual risks for ICH may be underestimated.

Besides an increased risk of VS-ICH, our findings show that antiplatelet therapy was associated with an increased hematoma volume, which is in line with previous data [6, 9]. In this regard, the primary objective of our study was to assess the risk of VS-ICH between endovascular and surgical treatment, as this is frequently debated in our clinical practice. While previous reports found no effect of VS-ICH on clinical outcome [3, 8, 15, 20], we chose not to analyze clinical outcome in our study because we do not believe that, in the context of a retrospective study, a meaningful correlation can be drawn between these largely minor hemorrhages and the overall clinical outcome in patients often severely affected by the subarachnoid hemorrhage. Regarding acute hemorrhage-related changes in clinical status, clinical evaluation was limited since most patients were sedated at the time of VS-ICH; however, no new oculomotor dysfunction was observed in any of the patients with VS-ICH in our study cohort.

Furthermore, this study carries all the limitations of a single-center retrospective analysis. Additionally, response to antiplatelet drugs was not monitored and antiplatelet dosing was not standardized. However, with respect to the number of ventriculostomies, this is the largest study so far investigating VS-ICH in endovascular and surgical patients suffering from subarachnoid hemorrhage.

In conclusion, endovascular treatment of ruptured aneurysms requiring antiplatelet drugs carries an increased risk of VS-ICH. This aspect needs to be considered when weighing endovascular and surgical treatment options.

## Data Availability

The data that support the findings of this study are available from the corresponding author upon reasonable request.
